# Case series of syphilis and HIV co-infections

**DOI:** 10.12669/pjms.293.3346

**Published:** 2013

**Authors:** Asrul Abdul Wahab, M.M. Rahman, Marlyn Mohammad, Salasawati Hussin

**Affiliations:** 1 Asrul Abdul Wahab, Department of Medical Microbiology & Immunology, Faculty of Medicine, The National University of Malaysia, Cheras 56000 Kuala Lumpur, Malaysia.; 2 M.M. Rahman, Department of Medical Microbiology & Immunology, Faculty of Medicine, The National University of Malaysia, Cheras 56000 Kuala Lumpur, Malaysia.; 3 Marlyn Mohammad, Department of Medical Microbiology & Immunology, Faculty of Medicine, The National University of Malaysia, Cheras 56000 Kuala Lumpur, Malaysia.; 4 Salasawati Hussin, Department of Medical Microbiology & Immunology, Faculty of Medicine, The National University of Malaysia, Cheras 56000 Kuala Lumpur, Malaysia.

**Keywords:** Co-infection, HIV, Penicillin, RPR, Syphilis

## Abstract

Syphilis and HIV co-infection are indeed dangerous combinations. The present communication describes three different cases of syphilis and HIV co-infection in young men. The first case is a 25-year-old medical graduate with a primary and secondary syphilis lesions at time of presentation. The second case is a 24-year-old government officer with right eye posterior uveitis where the serology tests for syphilis were reactive. His HIV tests were also positive. The final case is a 25-year-old homosexual who was admitted to the medical ward because of *Mycobacterium tuberculosis *infection. The HIV and syphilis serology tests were noted to be positive.

## INTRODUCTION

*Treponema pallidum subsp. pallidum *is the spirochete that causes syphilis. Untreated syphilis progress through several stages, which may lead to severe complications. The infection is mainly acquired through sexual contact although other modes of transmission have been reported.^1^ Previously, syphilis outbreaks often affected economically disadvantages minorities with poor access to health care, but recent study shows it is associated with sexual practice particularly among the homosexual population and those with human immunodeficiency virus (HIV) infection.^2^ The infection has also re-emerged in developed countries in homosexual population with high number of cases co-infected with HIV.^3^ Here, we discuss three cases of syphilis and HIV co-infection that occurred in young male patients with different risk factors and clinical manifestations. The study would help in managing similar patients with appropriate treatment.

## CASE REPORTS

**Case 1: **A 25-year-old medical graduate was diagnosed with HIV infection during investigation of the sharp injury. He presented to Infectious Disease Clinic for his scheduled appointment with complaint of chancre and rash over both hands and feet for one-week duration. His HIV infection was diagnosed about six months earlier. He had history of unprotected sexual intercourse with his newly met girlfriend for the last one month prior to this presentation.

On physical examination, he was afebrile. Lymphadenopathies were observed in his right and left cervical lymph nodes and presence of maculopapular rashes over his palms and soles together with chancre on his penis. No other abnormalities were observed.

The rapid plasma reagin (RPR) was reactive with the titer of 1: 128. The Syphilis IgM was also positive. The patient was treated with intramuscular Benzathine penicillin 2.4 mega units weekly for three doses. The follow up of RPR sample which was sent three months after completion of the treatment showed remarkable decrease in the titer to 1:4.

The patient was started on anti-retroviral therapy two months before the current presentation. The baselines CD4 and HIV viral load were 337 cells/μL and 328,000 copies/mL, respectively. During this follow-up, the CD4 count was 436cells/μL however, at that time no HIV viral load was done. The viral load at 6 months after the commencement of treatment showed 66 copies/mL, indicated the positive response of the treatment.

**Case 2: **A 24-year-old gentleman is a government officer, was referred to the ophthalmology clinic with blurring vision of his right eye for two weeks. The condition was also associated with glare and it was persistent in nature. There was no photophobia, red eye, eye itchiness, trauma or family history of connective tissue disorders. On further questioning, he was sexually active with his girlfriend. He had a history of painless penile ulcer about one year ago that resolved by itself without any treatment.

On examination, he was afebrile and right eye examination noted presence of posterior uveitis. No lymphadenopathy and body rash were observed.

Blood specimen was sent for serological investigation to determine the cause of uveitis. Serological test RPR was reactive at 1: 512 with positive for Syphilis IgG but negative for Syphilis IgM. HIV serology of the patient was positive and negative for toxoplasma. 

He was diagnosed to have posterior uveitis of the right eye secondary to syphilis. The patient was treated with intravenous penicillin 3 million units for 10 days. Following the treatment, his right eye’s vision was improved. The follow up RPR titer showed decrease in titer to 1:2.

**Case 3: **A 25 year-old male involved in homosexual activity with multiple partners, was admitted with the symptoms suggestive of pneumonia. He denied other high-risk behavior activity namely intravenous drug use and contact with person with tuberculosis.

On examination, he was febrile, hypotensive and tachypneic. The patient was dehydrated and looked cachexic. Oral candidiasis was seen. Lung examination noted presence of bibasal crepitations. There were lymphadenopathies at cervical and inguinal regions. His other systems were found to be normal.

Empirical treatment for tuberculosis was initiated after the microscopic examination of the sputum sample showing positive for acid-fast bacilli. Later, his sputum culture confirmed the presence of *Mycobacterium tuberculosis. *The treatment for tuberculosis was continued at standard dosages. In view of his high-risk behavior, several serological tests were done which included HIV and syphilis. His HIV test result was positive. RPR was also reactive with titer of 1:2. Both Syphilis IgM and IgG were positive, confirmed syphilis infection too. He was given intramuscular Benzathine penicillin 2.4 mega units weekly for three dosages. The repeated RPR titer after 15 months was 1:1. The repeated Syphilis IgM and IgG serology tests were negative.

## DISCUSSION

We have described three cases of syphilis and HIV co-infection in young men. All of them acquired syphilis by sexual contact. Both syphilis and HIV infections are part of sexually transmitted diseases. Sexual contact is one of the mode of transmissions for both infections, thus co-infection is perhaps common. In case 1, HIV was acquired not by a sexual contact but he was infected by syphilis via sexual contact. Patients in case 2 and 3 acquired both HIV and syphilis through sexual contact.

Recent outbreaks of syphilis have been reported in many parts of the world. Studies from developed countries like United States and United Kingdom have showed that increasing cases of syphilis were detected among men who had sex with men (MSM) and HIV-infected populations.^2^^,^^3^ In Asia, syphilis and HIV co-infection in China is also noted, a similar finding in which MSM is a high-risk population for both infection.^4^ Recent study from Brazil also describes the same finding.^5^ Perhaps, MSM is at high-risk population for syphilis and HIV co-infection. Our cases here showed that sexual behavior is perhaps related to the transmission of syphilis in HIV-infected persons but a large-scale study regarding syphilis acquisition among HIV population in this country will be useful.

Clinical manifestation of syphilis is not affected very much in HIV-infected populations. Thus, the manifestation is almost similar with those without HIV infection.^6^ However, there are some differences which can be seen in HIV-infected patients. In case one, we noted that the patient presented with both primary and secondary syphilis at the same time. This situation is observed more common in HIV-infected persons than those without HIV.^6^ In case 2, this patient was having uveitis secondary to syphilis infection. Although uveitis can occur at any stage of syphilis but it is demonstrated earlier among HIV-infected than those without HIV.^7^ Interestingly, syphilitic posterior uveitis, like in case 2, was found to occur more frequent in those concomitantly infected with HIV.^8^ Other differences include, HIV-positive patients tend to have more than one chancre, a larger and deeper primary lesion, higher rate of asymptomatic primary syphilis, more aggressive secondary syphilis and increase rate of early neurological involvement.^6^^,^^7^

Serological tests are the main laboratory investigation to confirm the diagnosis of syphilis infection. As co-infection is frequent, therefore, all patients with syphilis should also be screened for HIV infection and vice versa.^6^^,^^7^ However, serology limitation particularly among HIV-positive patients should be considered when interpreting syphilis serology results. In general, serology tests for syphilis can be divided into two categories namely non-treponemal antibody test and specific treponemal antibody test. The results of both tests may be affected in HIV-positive patients. The limitations include increased rate of negative serology test in both primary and secondary syphilis, increase false-negative non-treponemal antibody test due to prozone reaction, high rate of serological failure to clear non-treponemal antibody test after treatment and seroreversion to negative of specific treponemal antibody tests following treatment.^7^ In our cases here, we do not encounter any of the limitation for diagnosis of syphilis.

In our laboratory service, we use Rapid Plasma Reagin (RPR) titer as follow-up laboratory test in syphilis following the treatment. Serological failure can be defined as lack of 4-fold decreases in RPR titers at 13-months after treatment or 4-fold increase in RPR titers ≥ 30 days after treatment. Increased risk of serological failure has been reported to be more common among those with late stage of syphilis and HIV-infected patients.^9^ Serological failure was not seen in all of our cases here, however, we noticed that there was possible seroreversion of specific treponemal antibody test in case 3 because the follow-up sample at 15 months after the treatment was negative. The subsequent follow-up sample is needed for further confirmation of this condition. Furthermore, the determination of the immunocompromised state may be helpful to explain this phenomenon.

In case of syphilis and HIV co-infection, syphilis may also interfere with HIV management. Syphilis infection is associated with a significant increase in plasma HIV viral load and a significant decrease in CD4 cell counts.^10^ This phenomenon is mainly seen in primary and secondary syphilis. These levels returned to pre-syphilis levels or improved after syphilis treatment.^11^ This condition was not seen in our case 1 where the CD4 count is within normal limit and the HIV viral load was reduced dramatically following anti-retroviral therapy (ART). In case 2 and 3, patients were newly diagnosed with HIV infection at almost the same time as syphilis diagnosis thus ART was not yet started.

Treatment of syphilis in HIV-infected and non-HIV is similar. Treatment is given as appropriate for the stage of infection.^12^ Penicillin is the antibiotic of choice and it is recommended antibiotic in HIV-infected population because it can reach high concentration in central nervous system for treatment of neurosyphilis which is more common in this population.^7^ In our cases also, the treatment was given according to stage of syphilis and all patients recovered well after the treatment.

In conclusion, the importance of our cases is not only about syphilis and HIV co-infection but to highlight some of the differences in clinical manifestations and serological results of syphilis that might be important for management of such patients.
